# Supplementary figures and images for: Longitudinal analysis of the faecal microbiome in pigs fed Cyberlindnera jadinii yeast as a protein source during the weanling period followed by a rapeseed- and faba bean-based grower-finisher diet
Source: Anim Microbiome. 2022 Dec 9;4:62. doi: 10.1186/s42523-022-00217-5 (PMC9733224; doi:10.1186/s42523-022-00217-5)

# DADA2 read tracking summary

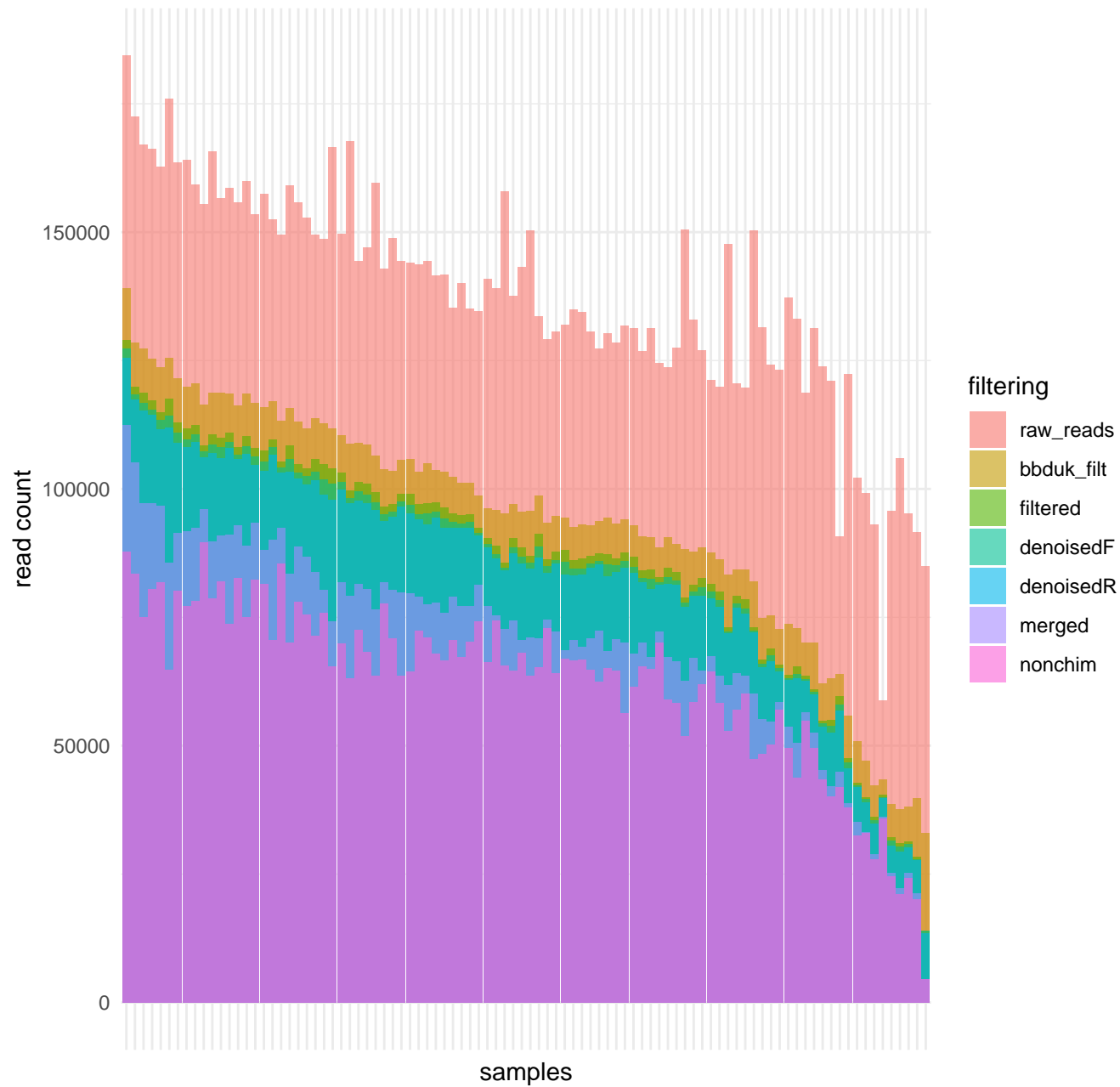

Supplement: Supplementary file 2 — Additional file 2: Figure S1. Read tracking summary. The bottom-most bars in the stack (nonchim) show the number of read that were the basis for making the feature count table (OTU/ASV-table). The bars above nonchim summarise the number of sequencing reads removed due to each procedure of the bioinformatics pipeline: (a) filtered with the bbduk filtering algorithm (bbduk filt), (b) filtered with the DADA2 algorithm (filtered), (c) removed due to DADA2 denoising procedure (denoisedR/F), (d) removed due to pair merging failures (merged). Raw reads are raw demultiplexed reads derived from Illumina sequencer. [file 42523_2022_217_MOESM2_ESM.pdf]

## Bacteroidetes

CL  
YL

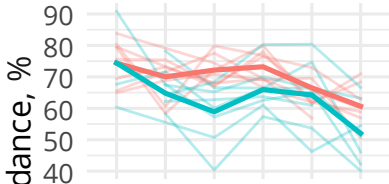

## Firmicutes

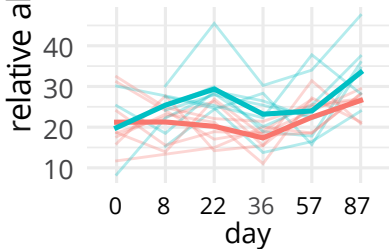

Supplement: Supplementary file 4 — Additional file 4: Figure S2. Relative abundance of Bacteroidetes and Firmicutes phyla across d0-88 PW. Individual observations are shown by the thin spaghetti lines, the average group values are shown by the thick spaghetti lines. [file 42523_2022_217_MOESM4_ESM.pdf]
